# Association between Sarcopenic Obesity and Arterial Stiffness in Korean Adults

**DOI:** 10.3390/jcm13206108

**Published:** 2024-10-14

**Authors:** Hye Rang Bak, Hye-Jin Jang, Hyun-Min Koh, Nak Gyeong Ko, Young Hye Cho

**Affiliations:** 1 Department of Family Medicine, Samsung Changwon Hospital, Sungkyunkwan University School of Medicine, Changwon 51353, Republic of Korea; hyerangbak@gmail.com (H.R.B.);; 2 Department of Family Medicine, Pusan National University School of Medicine, Yangsan 50612, Republic of Korea; 3 Department of Research & Support, Samsung Changwon Hospital, Sungkyunkwan University School of Medicine, Changwon 51353, Republic of Korea; 4 Department of Family Medicine and Biomedical Research Institute, Pusan National University Yangsan Hospital, Yangsan 50612, Republic of Korea

**Keywords:** sarcopenic obesity, arterial stiffness, cardiovascular risk, atherosclerosis

## Abstract

**Objectives**: This study examined the association between sarcopenic obesity and arterial stiffness using bioelectrical impedance analysis (BIA). **Methods**: This retrospective cross-sectional study included 20,601 Korean adults from January 2016 to December 2023. Sarcopenia was defined as height-adjusted appendicular skeletal muscle mass [(ASM/height^2^) <5.7 in women and <7.0 in men] using BIA. Obesity was defined by body mass index or waist circumference. Arterial stiffness was assessed by measuring brachial-ankle pulse wave velocity (baPWV). The participants were categorized into four groups: normal, sarcopenia, obesity, and sarcopenic obesity. The baPWV values were compared among the four groups to investigate the association between sarcopenic obesity and arterial stiffness using adjusted multivariate analyses. **Results**: The mean baPWV of the sarcopenic obesity group was higher (*p* < 0.001) than that of the other groups. The odds ratio for having high baPWV (>1800 cm/s) in the sarcopenic obesity group was 2.40 (95% CI, 1.07–5.38) after adjusting for age, sex, exercise, smoking, heavy alcohol consumption, hypertension, and dyslipidemia. **Conclusions**: Sarcopenic obesity was independently associated with increased arterial stiffness.

## 1. Introduction

Sarcopenia is characterized by a reduction in the quantity, quality, and functionality of skeletal muscles and is commonly linked to the natural process of aging [1]. There has been an increase in sarcopenia’s incidence owing to the growing global aging population. A recent meta-analysis included 151 studies and indicated a range of 10% to 27% in the prevalence of sarcopenia [2]. Moreover, there has been a significant rise in the prevalence of individuals with obesity. The World Obesity Atlas 2023 reported that the worldwide prevalence of overweight or obese individuals was 38% in 2020, with a projected increase of 51% by 2035 [3].

Sarcopenic obesity is characterized as the combination of sarcopenia and obesity, and as the number of individuals affected by both conditions rises, the prevalence of sarcopenic obesity is expected to increase as well. Consequently, there is a growing interest in the implications of negative clinical outcomes associated with sarcopenic obesity. In response to the reduction in muscle mass, there is an increase in adipose tissue, promoting inflammation and worsening insulin resistance, ultimately contributing to further muscle loss [4]. When one enters the vicious cycle of sarcopenia and obesity intertwined, it is referred to as sarcopenic obesity. Numerous research has shown that the combined presence of sarcopenia and obesity yields synergistic effects, resulting in sarcopenic obesity having more detrimental effects than either condition alone [5].

In particular, many clinical studies have shown that sarcopenic obesity has a greater impact on developing cardiovascular disease (CVD) and atherosclerosis than sarcopenia or obesity alone. Farmer et al. discovered that sarcopenic obesity was associated with the highest risk of CVD events compared with sarcopenia or obesity only [6]. Additionally, an analysis comparing the coronary artery calcium score (CAC) as a marker of atherosclerosis revealed that the sarcopenic obesity group had a significantly higher likelihood of having high CAC scores (≥100) compared to the other groups [7].

Arterial stiffness, measured by pulse wave velocity (PWV), is a commonly used indicator of atherosclerosis and CVD risk [8]. Brachial—ankle PWV (baPWV) provides a more accurate reflection of peripheral artery characteristics than carotid—femoral PWV (cfPWV). Thus, baPWV is regarded as a comprehensive measure of arterial stiffness, allowing precise assessment of CVD risk and risk stratification [9].

Previous studies have consistently shown strong correlations between arterial stiffness, sarcopenia, and obesity [10,11]. However, there is not enough clinical research on the association between sarcopenic obesity and arterial stiffness. The baPWV values of males were highest in the sarcopenic obesity group in the J-SHIPP study [12]. Additionally, a recent study utilizing cfPWV showed that the group with sarcopenic obesity exhibited the highest cfPWV values [13]. However, based exclusively on the evidence from these two studies, the association between sarcopenic obesity and arterial stiffness may be considered weak. Moreover, methodological limitations arise from variations in the definitions of sarcopenia and obesity, as well as differences in the indicators of arterial stiffness utilized in the two studies.

Therefore, our objective was to assess the association between sarcopenic obesity, as defined by the current guidelines, and baPWV. Furthermore, we investigated whether this association was independent of other CVD risk factors.

## 2. Materials and Methods

### 2.1. Study Population

This retrospective cross-sectional study analyzed data from adults aged ≥ 40 years at a university hospital in Korea from January 2016 to December 2023. We excluded individuals for the following reasons: CVD (angina, myocardial infarction, stent insertion, stroke, carotid stenosis); Diabetes mellitus (DM); cancer; chronic kidney disease (estimated glomerular filtration rate (eGFR) < 30 mL/min/1.73 m^2^); hepatic disease (hepatitis B or C, liver cirrhosis); overt thyroid dysfunction (thyroid stimulating hormone > 4.0 mU/L or free thyroxine (T4) > 1.8 µg/dL); steroid or sex hormone replacement therapy; mean ankle-brachial index (ABI) < 0.9; or missing values.

### 2.2. Clinical, Laboratory, and Anthropometric Measurements

All adult participants were required to complete a questionnaire before being interviewed by a physician. The questionnaire was used to obtain information regarding medical history, current treatment status, exercise, smoking, and alcohol consumption. DM was determined by a glycated hemoglobin (HbA1c) level ≥ 6.5%, a fasting plasma glucose (FPG) level ≥ 126 mg/dL, or the use of anti-diabetic medication. Systolic/diastolic blood pressure (BP) ≥ 140/90 mmHg or the presence of ongoing anti-hypertensive therapy was considered hypertension (HTN). Dyslipidemia (DL) was defined as low-density lipoprotein cholesterol (LDL-C) ≥ 130 mg/dL or taking cholesterol-lowering medications. The history of CVD, cancer, or liver cirrhosis was determined as per the physician’s diagnosis.

Exercise was categorized as moderate-to-severe intensity physical activity for >150 min/week. Smoking status was categorized into two distinct groups: smokers (ex- and current smokers) and nonsmokers (individuals who had never smoked). Heavy alcohol consumption was defined as >20 g/day for women and >30 g/day for men.

Height and weight were quantified utilizing an automated stadiometer (BSM330; Biospace, Seoul, Republic of Korea). Body mass index (BMI) was calculated by dividing the individual’s weight (kg) by the square of height (m^2^), and waist circumference (WC) was measured between the lowest ribs and the iliac crest of the pelvis. Systolic and diastolic BP were measured using an automatic BP monitor (EASY X 800; Jawon Medical Co., Seoul, Republic of Korea).

Venous blood samples were obtained after fasting for at least 8 h. FPG levels were measured with a glucose hexokinase assay (Glucose HK gen.3; Roche Diagnostics, Mannheim, Germany) using a Roche-Hitachi Cobas 8000 c702 analyzer (Roche Diagnostics). HbA1c levels were measured using liquid chromatography on a Tosoh HLC-723 G8 analyzer (Tosoh Co., Tokyo, Japan). Levels of various biomarkers, including total cholesterol, high-density lipoprotein cholesterol (HDL-C), LDL-C, triglyceride (TG), alanine aminotransferase (ALT), aspartate aminotransferase (AST), and serum high sensitivity-C-reactive protein (hs-CRP) were measured using the Cobas 8000 c702 analyzer (Roche Diagnostics). Creatinine levels were assessed using the Jaffe method [14]. The eGFR was calculated using the Modification of Diet in Renal Disease Study equation, which incorporates age, race, sex, and serum creatinine level.

### 2.3. Bioimpedance Analysis (BIA)

Appendicular skeletal muscle mass (ASM) was measured using the BIA Inbody 770 device manufactured by the Biospace Company (Seoul, Republic of Korea). During the test, participants were instructed to maintain a standing position for 5 min and grasp the handles of the analyzer, ensuring that both ends of their limb made contact with the electrodes.

ASM index (ASMI) was calculated using the following formula:

ASMI: Total limb lean muscle mass/(height)^2^, (kg/m^2^)

### 2.4. Definition of Sarcopenia, Obesity and Sarcopenic Obesity

ASM was measured using BIA to define sarcopenia. ASM was calculated as the sum of the lean muscle mass in both arms and legs. Sarcopenia was defined based on ASMI < 5.7 (women) and <7.0 (men), in accordance with criteria set by the Korean Working Group on Sarcopenia in 2022 [15] and the Asian Working Group for Sarcopenia (AWGS) in 2019 [16].

Obesity was defined as meeting the BMI or WC criteria recommended by the European Association for the European Society for Clinical Nutrition and Metabolism (ESPEN) and the Study of Obesity (EASO) 2022 consensus [17]: (1) BMI ≥ 25 kg/m^2^ according to the Asia-Pacific World Health Organization criteria [18]. (2) WC ≥ 85 cm (women), ≥90 cm (men), as suggested by the Korean Society for the Study of Obesity.

Sarcopenic obesity was defined by the presence of both low ASMI (<5.7 in women and <7.0 in men) and high BMI (≥25 kg/m^2^) or WC (≥85 cm in women and ≥90 cm in men).

### 2.5. baPWV

The baPWV was measured using an automatic device (VP-1000PLUS; Omron Healthcare, Kyoto, Japan). Participants were examined at rest in the supine position for at least 3 min, with blood pressure measuring bands placed on both the brachial and ankle. ABI was calculated bilaterally as the ratio of the ankle to the brachial systolic BP. The average of the left and right baPWV values was used for the analysis.

The baPWV assessment was categorized as normal (<1400 cm/s), borderline (1400–1800 cm/s), or abnormal (>1800 cm/s) [19]. The Korean Society of Hypertension 2018 guidelines consider baPWV > 1800 cm/s as an indicator of subclinical target organ damage [20]. Thus, the cutoff value for high baPWV in this study was established at 1800 cm/s.

### 2.6. Statistical Analysis

Continuous variables are presented as mean ± standard deviation, and categorical variables are presented as numbers and percentages. The participants were segmented into four subgroups based on ASMI and either BMI or WC: normal, sarcopenia, obesity, and sarcopenic obesity. We conducted an analysis of differences among the four subgroups using methods such as an analysis of variance or Pearson’s chi-squared test. Logistic regression analysis was conducted to examine the association between the sarcopenic obesity phenotype and baPWV. The odds ratio (OR) for having high baPWV (>1800 cm/s) was determined across the four distinct groups. The statistical software used in this study was Stata version 15.1 (Stata Corporation, College Station, TX, USA), and statistical significance was determined at *p* < 0.05.

## 3. Results

### 3.1. Baseline Characteristics Based on the Sarcopenic Obesity Phenotype

**Figure 1:**
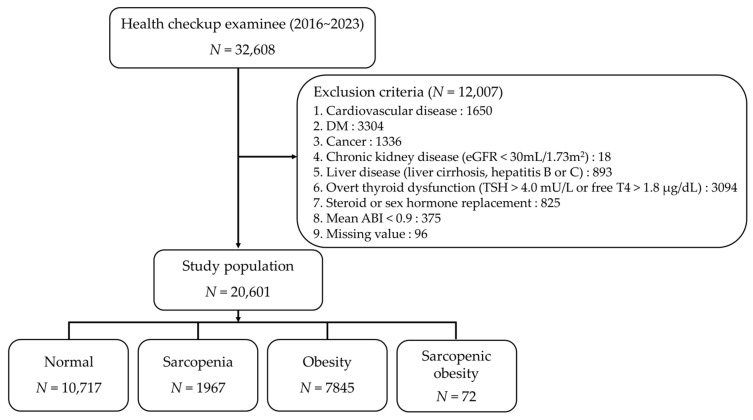
Study population (DM, diabetes mellitus; eGFR, estimated glomerular filtration rate; TSH, thyroid stimulating hormone; free T4, free thyroxine; ABI, ankle—brachial index).

We conducted an analysis of data from 32,608 health checkup examinees and excluded 12,007 individuals who met the exclusion criteria (Figure 1): CVD (1650), DM (3304), cancer (1336), chronic kidney disease (18), liver disease (893), overt thyroid dysfunction (3094), steroid or sex hormone replacement (825), lower ABI (375), and missing values (96). In total, 20,601 participants were categorized into four subgroups based on ASMI and either BMI or WC (Table 1): normal (control group; *n* = 10,717; 52.02%); sarcopenia (*n* = 1967; 9.54%); obesity (*n* = 7845; 38.08%); and sarcopenic obesity (*n* = 72; 0.34%). The mean ages of the sarcopenia and sarcopenic obesity groups were significantly higher. Additionally, the prevalence of HTN and DL was higher in the sarcopenic obesity group than in other groups.

We found the hs-CRP, a known predictor of CVD [21], elevated in the sarcopenic obesity group compared to the control group (mean ± s.d.: 2.02 ± 4.92 vs. 0.88 ± 2.42, *p* < 0.001). Additionally, differences were observed in the mean baPWV levels among the four subgroups (Table 1). In the sarcopenic obesity group, baPWV was the highest, while the sarcopenia group exhibited a higher baPWV than the obesity group (mean ± s.d.: 1321.03 ± 235.47, 1372.27 ± 252.80, 1340.49 ± 203.72, and 1434.09 ± 283.69 in order, *p* < 0.001) (obesity vs. sarcopenic obesity, *p* < 0.001).

### 3.2. ORs for Having High baPWV Based on the Sarcopenic Obesity Phenotype

Table 2 shows the association between sarcopenic obesity phenotype and high baPWV. We used a multivariable logistic regression analysis that adjusted for age, sex, exercise, smoking, heavy alcohol consumption, HTN, and DL. The ORs for having high baPWV remained significantly elevated following adjustment in the groups with sarcopenia, obesity, and sarcopenic obesity (multivariate adjusted OR [95% CI]: 2.19 [1.69–2.85]; 1.03 [0.83–1.27]; and 2.40 [1.07–5.38] in order; Table 2). Nevertheless, the association with obesity lost statistical significance following thorough full adjustments (Model 3: *p* = 0.805).

**Figure 2:**
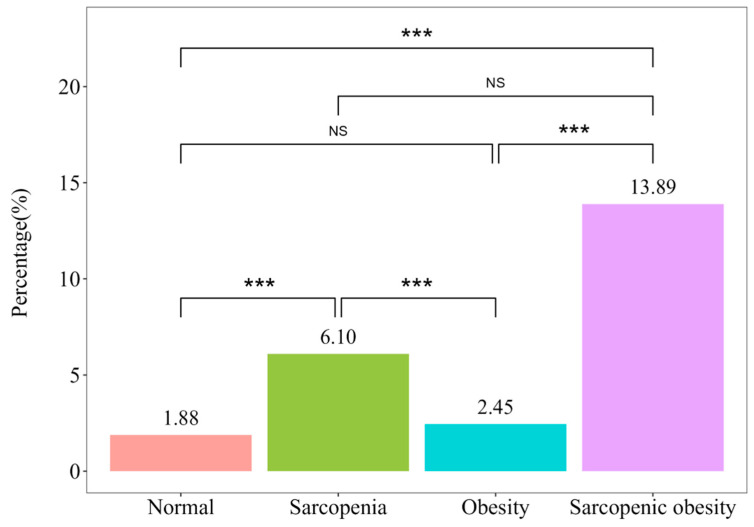
Prevalence of a high brachial—ankle pulse wave velocity based on the sarcopenic obesity phenotype (*** *p* < 0.001). NS = not significant.

There was a significantly higher prevalence of high baPWV in the sarcopenic obesity group compared to the control and obesity groups. However, non-significant differences were observed when compared with the sarcopenia group (percentages: 1.88%, 6.10%, 2.45%, and 13.89% in order; Figure 2).

## 4. Discussion

In this study, baPWV was the highest in the sarcopenic obesity group, and its value in the sarcopenia group was higher than that in the obesity group. This trend persisted while controlling for age, sex, lifestyle factors, and medical history. These results validate that sarcopenic obesity increases the risk of CVD, suggesting that the contribution of sarcopenia may be greater than that of obesity. According to our knowledge, this is not the first study to reveal that patients with sarcopenic obesity are more likely to have high baPWV. However, our study was conducted at a large scale and employed a different, broadly recognized definition of sarcopenic obesity.

According to previous studies, sarcopenic obesity is associated with negative clinical outcomes such as metabolic syndrome [22], DM [23], heart failure [24], non-alcoholic fatty liver disease [25], CVD [26], and mortality [27]. In this study, the individuals with sarcopenic obesity exhibited the highest rates of HTN and DL compared with those of the other groups, a pattern consistent with earlier research results (Table 1). Therefore, the loss of skeletal muscle and increase in fat mass may exacerbate their influence on cardiometabolic disorders [4].

In this study, baPWV values showed an increasing trend in the order of normal < obesity < sarcopenia < sarcopenic obesity (Table 1). Following adjustment for confounding variables as shown in Table 2, the probability of having high baPWV in comparison with the normal group exhibited similarities within the group of obesity (multivariate adjusted OR [95% CI]: 1.03 [0.83–1.27]); in contrast, the likelihood was 2.19 times higher in the sarcopenia group (2.19 [1.69–2.85]) and 2.4 times higher in the sarcopenic obesity group (2.40 [1.07–5.38]). The prevalence of high baPWV in each group also demonstrated a similar trend (Figure 2), with the prevalence in the sarcopenic obesity group being approximately twice that in the sarcopenia group (6.10% vs. 13.89%, *p* = 0.132). This indicates that there is an association between sarcopenic obesity and more increased arterial stiffness, which is explained by the combined influences of sarcopenia and obesity; in the relationship between sarcopenic obesity and arterial stiffness, the primary factor may be sarcopenia rather than obesity.

Our results are consistent with a J-SHIPP study that demonstrated an association between arterial stiffness and sarcopenic visceral obesity [12]. However, the J-SHIPP study focused on visceral obesity characterized by a visceral fat area (VFA) exceeding 100 cm², as measured by computed tomography. Additionally, sarcopenia was defined based on the cross-sectional area (CSA) of the thigh muscles adjusted for body weight (BW). Thus, the definition of sarcopenia used in the J-SHIPP study differs from that using international criteria. Nonetheless, unlike the J-SHIPP study, this study used definitions of obesity based on BMI or WC, which are commonly used in clinical practice, and sarcopenia based on the ASMI recommended by international guidelines. Therefore, our findings are important, even if they are the same as the results of previous studies. In addition, a study conducted by Fantin et al. found that the sarcopenic obesity group exhibited the highest cfPWV levels compared with the control, sarcopenia, and obesity groups [13]. Notably, this prospective study included 77 participants, with only 12 assigned to the sarcopenic obesity group, which may have limited the discriminatory power of the analysis.

Previous research has validated that sarcopenic obesity poses a higher CVD and atherosclerosis risk than obesity or sarcopenia alone, a finding that aligns with the results of our study. Farmer et al. discovered that sarcopenic obesity was associated with the highest risk of CVD events compared with sarcopenia or obesity only in no history of CVD (95% CI, 1.31–1.55, *p* = 0.532) [6]. In a study carried out by Park et al., there was no notable increase in the likelihood of a high CAC score (≥100) in the sarcopenia or obesity groups. However, the sarcopenic obesity group showed a significantly higher probability of a high CAC score (95% CI, 1.16–3.18, *p* = 0.011) [7]. Consequently, sarcopenic obesity is associated with a greater burden of atherosclerosis, indicating an increased risk of CVD development.

Sarcopenia and obesity are both well-known to be associated with arterial stiffness [10,28]. However, in our study, obesity did not show statistically significant ORs for having high baPWV after adjusting for disease history (Model 3: multivariate-adjusted OR [95% CI]: 1.03 [0.83–1.27], *p* = 0.805). Based on the research conducted by Hwang et al., adjustments for additional metabolic traits revealed a negative shift in the association between arterial stiffness and BMI, while the positive correlation with WC remained consistent [29]. Therefore, the association between obesity, as measured by BMI or WC, and arterial stiffness remains currently uncertain; therefore, including sarcopenia in conjunction with obesity may improve the assessment of CVD risk.

The mechanism by which sarcopenic obesity accelerates atherosclerosis is a complex process that is not fully understood; however, insulin resistance and inflammatory cytokines produced by the adipose tissue are major contributing factors [30]. Loss of skeletal muscle can lead to an increase in fat mass, and thereby, obesity promotes insulin resistance and inflammation [4]. Pro-inflammatory cytokines have the potential to cause mitochondrial dysfunction in skeletal muscle, disrupt insulin signaling, and lead to muscle atrophy. Thus, the interaction between sarcopenia and obesity exacerbates insulin resistance and creates a vicious cycle of metabolic deterioration. Additionally, this insulin resistance has an independent correlation with baPWV, an indicator of atherosclerosis [31]. Pro-inflammatory cytokines induced by sarcopenic obesity may lead to oxidative stress, which may worsen arterial stiffness, endothelial dysfunction, and microvascular damage, thereby increasing the risk of CVD [32]. Therefore, the underlying mechanism could be linked to oxidative stress triggered by insulin resistance and pro-inflammatory cytokines, resulting in arterial stiffness, emphasizing a significant association between sarcopenic obesity and atherosclerosis, ultimately causing CVD.

This study had several limitations. First, because this was a cross-sectional study, establishing a causal relationship between sarcopenic obesity and arterial stiffness was difficult. Second, whereas the 2019 AWGS defines sarcopenia based on muscle mass, strength, and function, our definition of sarcopenia focuses solely on muscle mass [16]. Although previous studies have shown a correlation between increased CVD risk and only low muscle mass [7,33], our results may not be sufficient to make comprehensive conclusions combining muscle strength and function. Third, our study found that sarcopenic obesity was present in 0.34% of participants, a lower rate than in previous research [34]. The reason is that this study was conducted in healthy individuals, and the use of BIA is also a critical factor. Previous research has shown that the prevalence of sarcopenic obesity is lower when using BIA than when using dual-energy X-ray absorptiometry [35]. As a result, the unusually low prevalence of sarcopenic obesity observed in this study may be challenging to apply to a larger demographic. Finally, participants at health screening centers may not fully represent the overall population. Therefore, the results of our study should be approached with caution.

## 5. Conclusions

This study demonstrated a significant association between sarcopenic obesity and arterial stiffness, as evidenced by an increased baPWV in the sarcopenic obesity group. Moreover, the sarcopenic obesity group showed elevated baPWV compared to the sarcopenia and obesity groups. Our findings indicate that there may be a synergistic interaction between sarcopenia and obesity, leading to the exacerbation of arterial stiffness. Furthermore, in the effect of sarcopenic obesity on arterial stiffness, sarcopenia appears to play a more critical role compared with that of obesity.

## Figures and Tables

**Table 1:** Baseline characteristics based on the sarcopenic obesity phenotype.

Characteristic	Total	Normal	Sarcopenia	Obesity	Sarcopenic Obesity	*p*-Value
	(*n* = 20,601)	(*n* = 10,717)	(*n* = 1967)	(*n* = 7845)	(*n*= 72)	
Sex						<0.001
Male	14594 (70.84)	7536 (70.32)	697 (35.43)	6339 (80.80)	22 (30.56)	
Female	6007 (29.16)	3181 (29.68)	1270 (64.57)	1506 (19.20)	50 (69.44)	
Age (y)	50.78 ± 6.94	50.66 ± 6.57	52.22 ± 8.49	50.56 ± 6.90	56.19 ± 10.36	<0.001
Anthropometric data						
Height (cm)	168.17 ± 8.02	168.37 ± 7.61	160.57 ± 7.32	169.88 ± 7.58	158.95 ± 8.17	<0.001
Weight (kg)	68.09 ± 11.69	64.12 ± 7.70	51.81 ± 6.16	77.66 ± 9.39	60.61 ± 5.04	<0.001
BMI (kg/m²)	23.96 ± 3.02	22.55 ± 1.57	20.08 ± 1.83	26.85 ± 2.20	24.04 ± 1.64	<0.001
Waist circumference (cm)	84.71 ± 8.45	80.70 ± 4.76	75.22 ± 5.18	92.53 ± 6.60	89.22 ± 5.36	<0.001
Body fat mass	17.30 ± 5.60	14.46 ± 3.35	13.72 ± 3.86	22.01 ± 5.14	23.34 ± 4.22	<0.001
Body fat mass (%)	25.35 ± 6.55	22.77 ± 5.67	26.43 ± 6.59	28.47 ± 6.06	38.59 ± 6.35	<0.001
Skeletal muscle mass	28.31 ± 5.73	27.64 ± 4.78	20.55 ± 3.45	31.26 ± 5.20	19.93 ± 3.51	<0.001
ASM (kg)	21.36 ± 4.52	20.94 ± 3.88	15.29 ± 2.98	23.51 ± 4.05	14.65 ± 2.85	<0.001
ASMI (kg/m²)	7.46 ± 1.05	7.32 ± 0.84	5.87 ± 0.67	8.08 ± 0.89	5.75 ± 0.66	<0.001
VFA (cm²)	84.35 ± 32.11	71.07 ± 22.77	66.73 ± 24.12	106.35 ± 30.73	145.17 ± 89.47	<0.001
Systolic BP (mmHg)	119.47 ± 11.63	117.76 ± 11.20	116.90 ± 12.49	122.43 ± 11.34	121.90 ± 12.46	<0.001
Diastolic BP (mmHg)	73.88 ± 9.39	72.54 ± 9.04	72.09 ± 9.29	76.15 ± 9.44	75.60 ± 8.82	<0.001
Biochemical markers						
Glucose (mg/dL)	95.47 ± 9.63	94.13 ± 9.28	92.79 ± 9.64	97.98 ± 9.55	95.29 ± 8.34	<0.001
HbA1c (%)	5.55 ± 0.33	5.51 ± 0.32	5.49 ± 0.33	5.61 ± 0.33	5.62 ± 0.39	<0.001
Total cholesterol (mg/dL)	201.45 ± 35.89	200.59 ± 34.91	202.17 ± 35.73	202.34 ± 37.11	212.14 ± 44.32	<0.001
LDL cholesterol (mg/dL)	132.26 ± 34.44	131.38 ± 33.51	128.56 ± 34.31	134.33 ± 35.51	139.26 ± 41.44	<0.001
HDL cholesterol (mg/dL)	59.42 ± 15.30	61.78 ± 15.43	68.62 ± 16.30	53.87 ± 12.70	60.00 ± 14.67	<0.001
Triglyceride (mg/dL)	129.43 ± 91.79	114.78 ± 75.20	97.55 ± 60.58	157.37 ± 110.03	136.85 ± 73.34	<0.001
AST (IU/L)	24.98 ± 14.07	23.81 ± 11.96	24.95 ± 20.62	26.54 ± 14.55	28.45 ± 13.98	<0.001
ALT (IU/L)	25.57 ± 17.81	22.51 ± 15.32	20.71 ± 15.41	30.93 ± 20.06	28.35 ± 18.71	<0.001
Creatinine (mg/dL)	0.89 ± 0.18	0.89 ± 0.17	0.77 ± 0.17	0.93 ± 0.18	0.73 ± 0.14	<0.001
hs-CRP (mg/L)	1.04 ± 2.95	0.88 ± 2.42	0.83 ± 2.57	1.30 ± 3.59	2.02 ± 4.92	<0.001
Co-morbidity						
Diabetes mellitus	0 (0.00)	0 (0.00)	0 (0.00)	0 (0.00)	0 (0.00)	-
Hypertension	4711 (22.87)	1732 (16.16)	302 (15.35)	2650 (33.78)	27 (37.50)	<0.001
Dyslipidemia	2690 (13.06)	1145 (10.68)	223 (11.34)	1304 (16.62)	18 (25.00)	<0.001
Health-related behavior						
Regular exercise	8545 (41.48)	4677 (43.64)	586 (29.79)	3265 (41.62)	17 (23.61)	<0.001
Smoking	4782 (23.21)	2392 (22.32)	242 (12.30)	2139 (27.27)	9 (12.50)	<0.001
Heavy alcohol consumption	3273 (15.89)	1693 (15.80)	176 (8.95)	1394 (17.77)	10 (13.89)	<0.001
Mean ABI	1.13 ± 0.09	1.13 ± 0.09	1.11 ± 0.07	1.13 ± 0.09	1.11 ± 0.09	<0.001
Mean baPWV (cm/s)	1333.73 ± 226.49	1321.03 ± 235.47	1372.27 ± 252.80	1340.49 ± 203.72	1434.09 ± 283.69	<0.001

Values are presented as mean ± standard deviation or number (%) by descriptive/frequency analysis. BMI, body mass index; ASM, appendicular skeletal muscle mass index; ASMI, appendicular skeletal muscle mass index; VFA, visceral fat area; BP, blood pressure; HbA1c, glycated hemoglobin; LDL, low-density lipoprotein; HDL, high-density lipoprotein; AST, aspartate aminotransferase; ALT, alanine aminotransferase; hs-CRP, high-sensitivity C-reactive protein; ABI, ankle—brachial index; baPWV, brachial—ankle pulse wave velocity. *p*-values were calculated using the analysis of variance or Pearson’s chi-squared test.

**Table 2:** Comparison of odds ratios for having high brachial—ankle pulse wave velocity based on the sarcopenic obesity phenotype.

	Model 1		Model 2		Model 3	
Variable	OR (95% CI)	*p*-Value	OR (95% CI)	*p*-Value	OR (95% CI)	*p*-Value
Normal	reference		reference		reference	
Sarcopenia	2.11 (1.63, 2.73)	<0.001	2.12 (1.64, 2.74)	<0.001	2.19 (1.69, 2.85)	<0.001
Obesity	1.26 (1.02, 1.55)	0.031	1.25 (1.01, 1.53)	0.037	1.03 (0.83, 1.27)	0.805
Sarcopenic obesity	2.85 (1.26, 6.46)	0.012	2.81 (1.24, 6.37)	0.014	2.40 (1.07, 5.38)	0.033

Values are presented as Odds ratio (95% confidence interval). OR, odds ratio; CI, confidence interval. Model 1: Adjusted for age and sex. Model 2: Adjusted for age, sex, exercise, smoking, heavy alcohol consumption. Model 3: Adjusted for age, sex, exercise, smoking, heavy alcohol consumption, HTN, and DL. *p*-values were calculated using the logistic regression analysis.

## Data Availability

Data are contained within the article.
